
JOURNAL INFORMATION
==============================
NLM Title Abbreviation: Lancet
Journal ID: Lancet
NLM Journal ID: 2985213R

Lancet (London, England)

ISSN: 0140-6736
EISSN: 1474-547X

ARTICLE INFORMATION
==============================
PMCID: PMC9950783
PMID: 36803433
DOI: 10.1016/S0140-6736(22)00045-9
Article ID: S0140-6736(22)00045-9
Article version: 1
Subjects: Department of Error

Department of Error

Print publication date: 2023 Feb 18
Volume: 401
Issue: 10376
First page: 556
Last page: 556
Copyright: © 2022 The Author(s). Published by Elsevier Ltd.
Copyright year: 2023
Copyright holder: Elsevier Ltd
License: This is an open access article under the CC BY license (http://creativecommons.org/licenses/by/4.0/).
License URL: https://creativecommons.org/licenses/by/4.0/

Erratum for: Trastuzumab deruxtecan versus trastuzumab emtansine in patients with HER2-positive metastatic breast cancer: updated results from DESTINY-Breast03, a randomised, open-label, phase 3 trial. 6 12 2022 Lancet 10.1016/S0140-6736(22)02420-5 36495879

==============================
Hurvitz SA, Hegg R, Chung W-P, et al Trastuzumab deruxtecan versus trastuzumab emtansine in patients with HER2-positive metastatic breast cancer: updated results from DESTINY-Breast03, a randomised, open-label, phase 3 trial. Lancet 2023; 401: 105–17—In figure 1 of this Article, the number at risk in the trastuzumab emtansine group at month 0 in panel B should have been 263, and the appendix has been updated. This correction has been made to the online version as of Feb 16, 2023.

